# The Effect of Statins on Bleeding in Isolated Coronary Artery Bypass Grafting Statins in CABG

**DOI:** 10.3390/jcm14238402

**Published:** 2025-11-27

**Authors:** Mustafa Karaarslan, Osman Fehmi Beyazal, Nihan Kayalar, Mehmed Yanartas

**Affiliations:** Department of Cardiovascular Surgery, Basaksehir Cam and Sakura City Hospital, Basaksehir G-434 Street No: 2L, Istanbul 34480, Turkey; karaarslanmustafa123@gmail.com (M.K.); nkayalar@hotmail.com (N.K.); myanartas@yahoo.com (M.Y.)

**Keywords:** coronary artery bypass grafting, statin, bleeding, atrial fibrillation, transfusion

## Abstract

**Background:** The aim of this study was to investigate the effect of preoperative statin use on postoperative bleeding and related complications in patients undergoing isolated coronary artery bypass grafting (CABG). **Methods:** Between 2023 and 2025, 627 patients who underwent isolated CABG were evaluated. The patients were divided into two groups: Group A (*n* = 241, received preoperative statins) and Group B (*n* = 386, did not receive preoperative statins). All preoperative, intraoperative parameters, and postoperative outcomes were compared. **Results:** Patient demographics, comorbidities, laboratory parameters, EuroSCORE II, echocardiographic findings, operative data, cross-clamp times, and cardiopulmonary bypass times were similar. Intraoperative and postoperative blood product use were comparable between the groups. Postoperative total bleeding was higher in Group A than in Group B, but no statistical difference was found. The postoperative exploration rate was higher in Group A than in Group B, but no statistically significant difference was found. There were no significant differences between the groups in terms of gastrointestinal bleeding. Postoperative atrial fibrillation (POAF) was significantly lower in Group A than in Group B (21 (8.7%)–74 (19.2%), *p* < 0.001). Mortality was higher in Group B than in Group A, but no statistically significant difference was found (3 (1.2%)–14 (3.6%), *p* = 0.07). Intensive care unit stay was longer in Group B than in Group A. A significant negative association was found between statin usage and POAF (*p* = 0.001, OR = 0.418). **Conclusions:** We found no statistically significant increase in postoperative bleeding or blood product use with preoperative statin therapy in isolated CABG patients. However, we found that preoperative statin therapy was protective against POAF.

## 1. Introduction

Bleeding is a major cause of morbidity and mortality following cardiac surgery, affecting around 15% of cases [1]. The use of blood products can lead to complications related to transfusions and higher medical costs [2]. Various factors, such as the type of cardiac surgery, medications administered, the use of cardiopulmonary bypass (CPB), and patient-specific risk factors, can all play a role in causing bleeding. By addressing and modifying these risk factors, it is possible to decrease bleeding and ultimately reduce mortality rates.

According to the 2023 ESC Guidelines for the Management of Acute Coronary Syndromes, high-dose statin therapy is recommended to be initiated and maintained as early as possible, regardless of baseline low-density lipoprotein cholesterol (LDL-C) [3]. However, statins have pleiotropic and antithrombotic effects, and given their cholesterol-lowering benefits, the clinical significance of these effects on the cardiovascular system remains controversial [4]. It remains unclear whether preoperative statin use increases bleeding and related complications following cardiac surgery. Due to these effects, there are concerns about their potential adverse effects on bleeding, but there is a lack of sufficient detailed studies. Therefore, we designed this study in coronary artery bypass graft (CABG) patients, one of the patient groups in which statins are most frequently used. The aim of this study was to investigate the effect of preoperative statin use on postoperative bleeding and related complications in CABG patients.

## 2. Methods

This retrospective, single-center, observational study included 627 patients. All patients aged 18 years and over who underwent isolated CABG at the Cardiovascular Surgery Clinic of Istanbul Basaksehir Cam and Sakura City Hospital between January 2023 and September 2025 were included in the study. Patients who had non-CABG procedures, concomitant procedures with CABG, those who did not use CPB, reoperations, those who used perioperative intra-aortic balloon pump (IABP) and extracorporeal membrane oxygenation (ECMO), and those who underwent dual antiplatelet therapy were excluded from the study. The patients were divided into two groups: Group A (*n* = 241, who received preoperative statins) and Group B (*n* = 386, who did not receive preoperative statins).

All patients’ medical records, demographic characteristics, comorbidities, preoperative and postoperative first-week transthoracic echocardiography (TTE) findings, preoperative and postoperative first-day laboratory parameters, surgical procedure details, cross-clamp (XCL) times, CPB times, vasoactive inotrope scores (VIS), EuroSCORE II, bleeding amount, blood product use, postoperative complications (postoperative exploration, postoperative atrial fibrillation (POAF), cerebrovascular accident (CVA), continuous renal replacement therapy (CRRT) need, sternal wound infection (SWI), gastrointestinal bleeding, intubation time, hospital and intensive care unit (ICU) stay, and mortality were recorded. Groups A and B were compared for all these parameters.

This study was approved by the Istanbul Basaksehir Cam and Sakura City Hospital Ethics Committee (Decision no: 2025-375). The study was conducted in accordance with the Declaration of Helsinki. Artificial intelligence-assisted technologies were not used in the production of the submitted work.

## 3. Statistics

The data were analyzed with SPSS software version 20.0 (IBM, Armonk, NY, USA). Continuous variables are presented as the minimum, maximum, median, and interquartile range. Categorical variables are expressed as numbers and percentages. The normality of data distributions was assessed using the Kolmogorov–Smirnov test. For numerical variables, differences between the patients and controls were tested using the *t*-test for parametric data or the Mann–Whitney U test for nonparametric data. Categorical variables were analyzed using the Pearson χ^2^ test and Fisher’s exact test for parametric and nonparametric data, respectively. Multivariate logistic regression analysis was performed to determine the factors affecting POAF. The level of statistical significance was set at *p* < 0.05.

## 4. Results

The patient’s demographics, comorbidities, TTE findings, and operative data are presented in Table 1. The mean age was 60.1 ± 8.8 years, with 119 (19%) female patients. The mean follow-up period was 404.4 ± 236 days (median: 393, 1–998 days). There was no difference between the groups in terms of age, height and gender. Hypertension (HT) and diabetes mellitus (DM) were more common in Group A than in Group B. Other comorbidities and VIS were also similar between the groups. There was no statistically significant difference between the groups in terms of EuroSCORE II values (mean 1.9–2.3, respectively). Preoperative and postoperative ejection fraction (EF) and tricuspid annular plane systolic excursion (TAPSE) values were also similar between the groups. There were no significant differences between the groups in terms of emergency surgery rates, coronary endarterectomy rate, left internal thoracic artery use, graft count, CPB times, and XCL times.

Comparisons of bleeding volume, blood product use, and intraoperative medication results are presented in Table 2. Intraoperative and postoperative red blood cells (RBCs), fresh frozen plasma (FFP), and apheresis platelet suspension (APS) use were similar between the groups. Postoperative day 1 bleeding was higher in Group A than in Group B, but no statistical difference was found (mean, 450 mL–400 mL, respectively, *p* = 0.16). Similarly, postoperative total bleeding was higher in Group A than in Group B, but no statistical difference was found (mean, 750 mL–700 mL, respectively, *p* = 0.09). There was no difference between the groups in terms of preoperative and postoperative activated clotting time (ACT) values. The dosages of intraoperative tranexamic acid and fibrinogen concentrate were also similar between the groups. Comparisons of preoperative and postoperative laboratory parameters are presented in Table 3. Preoperative and postoperative white blood cells, hematocrit, platelet, creatinine, sodium, and potassium values were similar between the groups. Only low-density lipoprotein was lower in Group A than in Group B (median 87 mg/dL–116 mg/dL, respectively, *p* < 0.001).

A comparison of postoperative outcomes is presented in Table 4 and Table 5. The postoperative exploration rate was higher in Group A than in Group B, but no statistically significant difference was found (15 (6.2%)–17 (4.4%), respectively, *p* = 0.39). There were no significant differences between the groups in terms of CVA, CRRT need, SWI, and gastrointestinal bleeding. POAF was significantly lower in Group A than in Group B (21 (8.7%)–74 (19.2%), respectively, *p* < 0.001). Mortality was higher in Group B than in Group A, but no statistically significant difference was found (3 (1.2%)–14 (3.6%), respectively, *p* = 0.07). No significant differences were found between the groups in terms of intubation time and hospital stay. However, ICU stay was longer in Group B than in Group A (mean 2.6 days–2.9 days, respectively, *p* = 0.04).

The results of multivariate regression analysis for factors affecting POAF are presented in Table 6. Accordingly, no significant association was found between gender, DM, HT, and POAF. However, a significant association was found between age and POAF (*p* < 0.01, OR = 1.091 [95% CI, 1.060–1.124]). Additionally, a significant negative association was found between statin usage and POAF (*p* = 0.001, OR = 0.418 [95% CI, 0.245–0.712]).

## 5. Discussion

Hydroxymethylglucoenzyme A (HMG-CoA) reductase inhibitors, commonly known as statins, play an important role in the treatment of cardiovascular disease. In addition to lowering lipids, they also exhibit antithrombotic and pleiotropic effects through various mechanisms. Statins increase the activity of endothelial nitric oxide synthase (eNOS) [5]. Pravastatin has been shown to reduce the expression of platelet membrane RhoA and tissue factor (TF) protein [6]. Atorvastatin and simvastatin decrease platelet activity by reducing the synthesis of thromboxane A2 (TXA2) [7]. They achieve this by inhibiting TXA2 synthesis through the blockade of platelet phospholipase A2 (PLA2) phosphorylation and the MAP kinase pathway [8]. Atorvastatin and simvastatin lower platelet activity by exposing CD36 and LOX-1 before significant LDL reduction, resulting in a direct antiatherothrombotic effect [9]. Statins alter the balance of the tissue plasminogen activator/plasminogen activator inhibitor (tPA/PAI-1) ratio in favor of fibrinolysis [10]. Simvastatin inhibits thrombin generation and factor V activation [11]. Fluvastatin, when administered within 6 h of acute coronary syndrome, reduces soluble endothelial protein C receptor (sEPCR), thereby decreasing thrombin activation [12]. Early administration of atorvastatin in normocholesterolemic patients with unstable angina significantly affects von Willebrand factor (vWF) levels and liver-derived components of the thrombosis and fibrinolysis systems [13]. Pravastatin regulates thrombomodulin (TM) expression by inhibiting Rac1 and Cdc42 activation and NF-kappaB [14]. Kruppel-like factor 2 (KLF2) has anticoagulant and atheroprotective effects by up-regulating thrombomodulin and eNOS and down-regulating TF and PAI-1, with statins playing a role in upregulating KFL2 [15]. All of these effects raise concerns that statins may increase bleeding after cardiac surgery. However, no comprehensive studies have been conducted on this topic. Therefore, we designed this study to investigate the effect of statins on bleeding after cardiac surgery.

In addition to this, numerous studies have demonstrated the beneficial effects of statins on the cardiovascular system. Statins stabilize atherosclerotic plaque and reduce the incidence of thrombotic events such as myocardial infarction and stroke. Furthermore, statins have anti-inflammatory and antioxidant effects in cardiac surgery patients [16]. In addition to their cholesterol-lowering effects, they play an important role in the primary and secondary prevention of cardiovascular events. The benefits of statins for saphenous vein graft patency are well known [17]. Therefore, perioperative statins are widely used in patients undergoing CABG. Furthermore, due to similar pathophysiological processes, they are frequently used in peripheral artery disease and carotid stenosis. Statins have also been reported to be protective against deep vein thrombosis [18]. However, there are also concerns about their effects on bleeding and some studies have been reported on this. Goldstein et al., in a randomized trial of patients with cerebrovascular disease, reported an increased risk of hemorrhagic stroke in patients taking high-dose simvastatin compared to placebo, although not statistically significant [19]. Martinez et al. also reported that, in a population aged 30–65 years, statin users had a higher risk of gastrointestinal bleeding than other chronic medication users [20]. However, it is unclear whether statin use increases bleeding in cardiac surgery patients. Our study, however, found no clinically significant increase in bleeding and related complications in isolated CABG patients.

To investigate the effect of statins on bleeding, we established a rigorous exclusion criterion and attempted to ensure that similar risk factors for bleeding were present. We excluded patients except for isolated CABG patients to avoid the influence of factors related to the operation characteristics on the results. Indeed, the patient demographics, all comorbidities except diabetes and hypertension, all laboratory parameters except LDL-c, EuroSCORE II, TTE findings, all operative data, XCL times, and CPB times were similar. The lower LDL-c values in Group A indicate effective statin use. Indeed, the median LDL-c value was 116 mg/dL in Group B, as patients were not using statins. In this regard, we found that all intraoperative and postoperative blood product use was similar between the groups. Despite similar ACT values in the postoperative period, postoperative day 1, and total bleeding amounts were higher in Group A, without a statistically significant difference. This difference was not clinically significant either. Indeed, postoperative exploration and gastrointestinal bleeding rates were also higher in Group A, but these were not statistically significant. Nenna et al. reported that preoperative atorvastatin use was associated with a reduction in bleeding and blood product use after CABG [21]. Wu et al. also reported a reduction in the risk of major bleeding with concurrent statin use in patients with non-valvular atrial fibrillation (AF) receiving direct oral anticoagulants [22]. Powell et al. found no difference in reoperation due to bleeding between patients receiving lipid-lowering therapy and those not receiving it in CABG patients [23]. Antoniou et al. reported an increased risk of major bleeding with simvastatin and lovastatin in elderly patients receiving Dabigatran [24]. In our study, although there was a numerical difference between the groups in terms of bleeding, it was not statistically significant. We can attribute this non-significant increase to all the antithrombotic and pleiotropic effects mentioned above. However, this results suggest that these effects are clinically insignificant, and statins are not associated with a significant increase in bleeding and related complications. However, it is clear that prospective studies are needed to prove that there is no adverse effect on bleeding in cardiac surgery.

Another important point regarding statins is their effects on other postoperative outcomes. A meta-analysis by Putzu et al. reported that statins did not protect against stroke and infection [25]. However, statin therapy has been shown to reduce the risk of stroke, and interventions specific to the dyslipidemic profile have been shown to reduce the risk of CVA [26]. In our study, we found no difference between the groups in terms of CVA and SWI. Additionally, Layton et al. demonstrated that preoperative statin therapy reduced acute kidney injury (AKI) after cardiac surgery [27]. Zhao et al. reported that perioperative statin therapy did not reduce the risk of AKI in patients undergoing cardiac surgery [28]. Similarly, we found no significant difference between the groups in terms of CRRT requirement. Vaduganathan et al. reported that preoperative statin therapy reduced mid-term mortality after cardiac surgery, independent of baseline lipid levels [29]. In our study, mortality was lower in the statin group compared to those not taking the statin, although statistically not significant (*p* = 0.07). This result may be due to the protective effect of statins, or it may be related to the lower EuroSCORE II in Group A. Khun and colleagues reported that preoperative statin treatment reduced ICU and hospital stays [30]. In our study, there was no difference between the groups in terms of intubation time and hospital stay. We only observed that ICU stays were shorter in those receiving statins. We believe this is primarily due to POAF, the only significant difference between the groups. More frequent POAF may lead to longer ICU stays. Statin use may indirectly help reduce ICU stay.

Moreover, there have been numerous studies conducted on the effects of statins on POAF. Sakamoto et al. reported that preoperative statin therapy reduced the incidence of AF after CABG [31]. Aydin et al. also found that postoperative statin therapy decreased the likelihood of new-onset AF after isolated CABG [32]. However, Zheng et al. reported that preoperative statin therapy did not prevent the development of postoperative AF in patients undergoing elective cardiac surgery [33]. In our study, we observed that POAF occurred significantly less frequently in patients who received preoperative statins compared to those who did not. Additionally, our multivariate analysis revealed that statin use independently protected against POAF (OR = 0.418). These results suggest that statins may have a protective effect against POAF. Inflammation plays an important role in the pathophysiology of POAF and the antioxidant and anti-inflammatory effects of statins may be the reason why POAF is less common in patients taking statins [32]. By reducing POAF, statins contribute to both a reduction in ICU stays and may help reduce hospital stays. By reducing the risk of neurological complications associated with POAF, statin use may further contribute to reducing morbidity and mortality in the medium to long term. Nonetheless, these findings should be validated in future prospective randomized controlled studies.

Statins are recommended by guidelines for their beneficial effects on the cardiovascular system and are widely used for CABG. Although their antithrombotic effects raise concerns about bleeding and related complications, we did not observe any adverse effects on clinical outcomes in this study regarding bleeding. Therefore, we think that statins should be used before and after CABG due to their known beneficial effects on the cardiovascular system and their potential protective effects against POAF. Despite being a higher-risk cohort, Group A had significantly less POAF and a trend toward lower mortality. The use of statins in the pre- and postoperative period for CABG patients is extremely important and necessary to improve postoperative outcomes.

## 6. Limitations

The first limitation of this study is that it is retrospective, single-center, and not a controlled study. However, this study did not differentiate between subtypes, preventing a comparison. Future investigations with different active ingredients would be beneficial. Third, the effects of the drugs may vary depending on the duration of use. The effects could differ between patients who have been taking statins for a long time and those who were newly started on them preoperatively. Additionally, different doses of statins may have different effects, but we were unable to make this comparison in this study. We believe that comparing different doses is necessary in the future. This study did not examine the number of days patients had been taking statins before surgery. Further research on this aspect could provide valuable insights. This study was conducted in isolated CABG patients and it would be useful to investigate it in other cardiac surgical procedures in the future. This small, non-statistically significant difference in bleeding and associated octomes was underpowered due to the retrospective design of the study. Future, more detailed prospective studies may clarify this issue.

## 7. Conclusions

**Figure 1:**
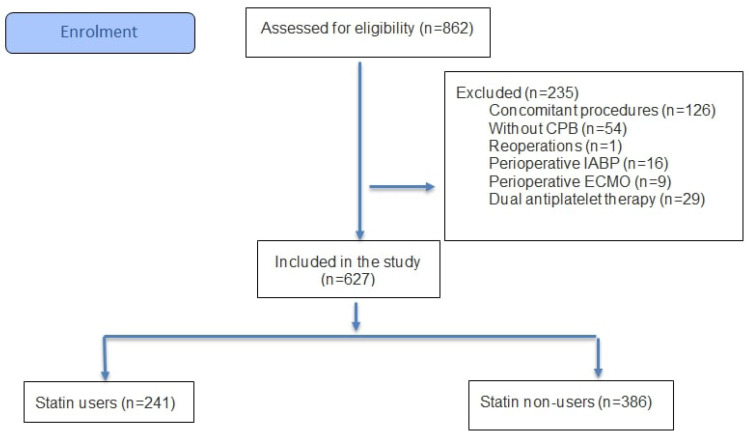
Flow diagram for study participants.

In this retrospective study, we found no statistically significant increase in postoperative bleeding or blood product use with preoperative statin therapy in isolated CABG patients. However, we found that preoperative statin therapy was protective against POAF. Future large randomized controlled trials are needed to investigate this topic further (Figure 1).

## Figures and Tables

**Table 1:** Comparison of patient demographics, comorbidities, medications, echocardiographic findings, and operative data between Group A and Group B.

	Group A (*n* = 241) Statin (+)			Group B (*n* = 386) Statin (−)			
	Min-Max or *n* (%)	Median (Mean)	IQR	Min-Max or *n* (%)	Median (Mean)	IQR	*p*
**Demographic data**							
Gender female	47 (19.5)			72 (18.7)			0.79
Age (years)	38–84	60	10	34–86	61	14	0.34
Height (cm)	137–183	170	12	135–196	169	10	0.73
Weight (kg)	53–127	80	18	45–123	79.5	17	0.15
Body surface area (kg/m^2^)	1.44–2.42	1.91	0.21	1.26–2.42	1.89	0.21	0.22
Body mass index (m^2^)	18.7–55.6	28.3	5.7	19.6–42.8	27.7	5.6	0.14
**Comorbid diseases**							
Diabetes Mellitus	140 (58.1)			192 (49.7)			**0.04**
Hypertension	171 (71)			216 (56)			**<0.001**
Chronic obstructive pulmonary disease	20 (8.3)			27 (7)			0.54
Cerebrovascular accident	11 (4.6)			28 (7.3)			0.17
Preoperative atrial fibrillation	2 (0.8)			3 (0.8)			0.63
Chronic renal failure	12 (5)			18 (4.7)			0.85
Peripheral artery disease	14 (5.8)			24 (6.2)			0.83
Thyroid disorder	13 (5.4)			30 (7.8)			0.25
EuroSCORE II	1–13	1 (1.9)	1	1–42	1 (2.3)	1	0.17
Vasoactive inotropic score (VIS)	0–70	0 (3.6)	4	0–440	0 (5.7)	5	0.56
**Echocardiographic findings**							
Preop ejection fraction (%)	22–65	60	10	20–65	55	10	0.17
Postop ejection fraction (%)	14–32	23	5	14–33	23	4	0.28
Preop TAPSE	20–65	55	10	25–65	55	10	0.25
Postop TAPSE	11–29	16	6	9–31	17	4	0.51
**Operative data**							
Emergency surgery	8 (3.3)			24 (6.2)			0.10
Coronary endarterectomy	12 (5)			19 (4.9)			0.97
LITA usage	218 (90.5)			336 (87)			0.19
The number of grafts	1–6	3 (3.1)	1	1–7 (3.1)	3	1	0.21
Cross-clamp time (min)	26–201	76	46	22–217	81	46	0.14
Cardiopulmonary bypass time (min)	52–268	123	50	40–407	130	54	0.11

IQR: interquartile range, EuroSCORE II: The European System for Cardiac Operative Risk Evaluation, TAPSE: tricuspid annular plane systolic excursion, LITA: left internal thoracic artery; bold values indicate statistical significance.

**Table 2:** Comparison of bleeding amounts, blood products, and intraoperative medications between Group A and Group B.

	Group A (*n* = 241) Statin (+)			Group B (*n* = 386) Statin (−)			
	Min-Max or *n* (%)	Median (Mean)	IQR	Min-Max or *n* (%)	Median (Mean)	IQR	*p*
**Blood products and bleeding amounts**							
Intraop red blood cells using	0–6	0 (0.78)	1	0–13	0 (0.83)	2	0.23
Intraop fresh frozen plasma using	0–4	0 (0.27)	0	0–3	0 (0.21)	0	0.30
Intraop platelet suspensions	0–2	0 (0.04)	0	0–2	0 (0.06)	0	0.33
Postop red blood cells using	0–8	0 (0.91)	1	0–16	0 (0.98)	1	0.96
Postop fresh frozen plasma using	0–7	0 (0.75)	1	0–9	0 (0.75)	1	0.99
Postop platelet suspensions	0–2	0 (0.07)	0	0–6	0 (0.08)	0	0.15
Postop 1st day amount of bleeding (ml)	50–2100	450 (512)	300	50–1725	400 (481)	300	0.16
Postop total amount of bleeding (ml)	200–2950	750 (827)	350	100–3025	700 (782)	450	0.09
**Intraoperative medications**							
Preoperative ACT	79–256	126 (130)	40	56–357	126 (132)	38	0.81
Postoperative ACT	86–237	123 (125)	22	59–240	125 (128)	27	0.08
Dose of tranexamic acid (mg)	0–3000	1000 (788)	1000	0–3000	1000 (827)	1000	0.48
Dose of fibrinogen concentrate (mg)	0–4000	0 (414)	500	0–4000	0 (318)	0	0.11

ACT: activated clotting time; bold values indicate statistical significance.

**Table 3:** Comparison of laboratory parameters between Group A and Group B.

	Group A (*n* = 241) Statin (+)		Group B (*n* = 386) Statin (−)		
	Min-Max or *n* (%)	Median (IQR)	Min-Max or *n* (%)	Median (IQR)	*p*
**Preop laboratory parameters**					
White blood cells (10^9^/L)	3.2–18.6	8.3 (2.8)	2–19.4	8.6 (3)	0.37
Hematocrit (%)	24–52	40.9 (5.6)	20.5–52.5	41.3 (6.1)	0.10
Platelets (10^9^/L)	112–586	249 (77)	63–565	253 (99)	0.40
Creatinine (mg/dL)	0.27–9.02	0.95 (0.30)	0.42–11.8	0.91 (0.29)	0.06
Sodium (mEq/L)	127–146	139 (4)	124–150	139 (4)	0.64
Potassium (mEq/L)	3.4–6	4.37 (0.62)	2.96–6.37	4.36 (0.6)	0.69
Low-density lipoprotein (mg/dL)	31–243	87 (48)	24–377	116.5 (57)	**<0.001**
**Postop 1st day laboratory parameters**					
White blood cells (10^9^/L)	7.2–44.2	16.3 (8.1)	5.3–50.3	16.5 (7.9)	0.63
Hematocrit (%)	20.5–37.5	28 (5.4)	19.8–60	28.4 (5.1)	0.36
Platelets (10^9^/L)	53–385	179 (75)	42–496	190 (82)	0.06
Creatinine (mg/dL)	0.08–5.38	1.2 (0.46)	0.04–7.7	1.18 (0.41)	0.29
Sodium (mEq/L)	135–155	143 (5)	133–153	142 (4)	0.52
Potassium (mEq/L)	2.75–5.76	4.22 (0.69)	2.6–5.76	4.31 (0.68)	0.14

Bold values indicate statistical significance.

**Table 4:** Comparison of postoperative outcomes between Group A and Group B.

	Group A (*n* = 241) Statin (+)	Group B (*n* = 386) Statin (−)	*p*
Postoperative exploration	15 (6.2)	17 (4.4)	0.39
Cerebrovascular accident	2 (0.8)	9 (2.3)	0.16
Continuous renal replacement therapy	4 (1.7)	9 (2.3)	0.56
Postop atrial fibrillation	21 (8.7)	74 (19.2)	**<0.001**
Deep sternal wound infection	11 (4.6)	14 (3.6)	0.56
Gastrointestinal bleeding	2 (0.8)	1 (0.3)	0.57
Mortality	3 (1.2)	14 (3.6)	0.07

Bold values indicate statistical significance.

**Table 5:** Comparison of intubation time and hospital stay between Group A and Group B.

	Group A (*n* = 241) Statin (+)			Group B (*n* = 386) Statin (−)			
	Min-max or	Median (Mean)	IQR	Min-max or	Median (Mean)	IQR	*p*
Intubation time (hours)	1–240	10	7	2–672	9	6	0.09
Intensive care unit stay (days)	1–22	2 (2.6)	1	1.28	2 (2.9)	1	**0.04**
Hospital stay (days)	2–104	6	3	1–64	7	3	0.25

Bold values indicate statistical significance.

**Table 6:** Multivariate regression analysis for factors affecting postoperative atrial fibrillation.

	Odds Ratio	CI % 95	*p*
Gender female	0.597	0.311–1.147	0.12
Age	**1.091**	1.060–1.124	**<0.001**
Diabetes Mellitus	0.779	0.476–1.274	0.31
Hypertension	1.085	0.650–1.813	0.75
Statin usage	**0.418**	0.245–0.712	**0.001**

Bold values indicate statistical significance.

## Data Availability

The data supporting this study are available upon reasonable request for research purposes.
